# Novel observations of Pacinian corpuscle distribution in the hands and feet based on high-resolution 7-T MRI in healthy volunteers

**DOI:** 10.1007/s00256-020-03667-7

**Published:** 2020-11-06

**Authors:** Christoph Germann, Reto Sutter, Daniel Nanz

**Affiliations:** 1 Radiology, Balgrist University Hospital, Forchstrasse 340, CH-8008 Zurich, Switzerland; 2 University of Zurich, Zurich, Switzerland; 3 SCMI, Swiss Center for Musculoskeletal Imaging, Balgrist Campus AG, Zurich, Switzerland

**Keywords:** Pacinian corpuscle, Vater-Pacini, Lamellar corpuscle, Mechanoreceptor, MRI, 3D imaging, Hand, Foot

## Abstract

Pacinian corpuscles represent special nerve endings that serve as mechanoreceptors sensitive to vibration and pressure and are crucial for proprioception. This work demonstrates that the complex network of Pacinian corpuscles in hands and feet can be examined with three-dimensional Dual Echo Steady State (DESS) MR imaging at 7 T, while previous dedicated MRI reports were either limited to two-dimensional images or focused on the hands. The high-resolution MR images show the detailed architecture of the complex receptor network and reveal a “chain-like” arrangement of Pacinian corpuscles, a predilection for clustering around metacarpophalangeal/metatarsophalangeal joints, proximal phalanges and fingertips, and specific sensor locations both in the superficial subcutaneous tissue and adjacent to deep soft tissue structures such as tendons and joint capsules.

## Introduction

Pacinian corpuscles, also known as Vater-Pacini or lamellar corpuscles, are sensory receptors for vibration and deep pressure and are essential for proprioception [1]. They can be found throughout the whole body; however, it is the hands and feet where they occur most numerously and tightly grouped. Pacinian corpuscles have been found to be located adjacent to and within capsuloligamentous structures where they can provide reflexogenic feedback and offer indirect support regarding joint stability [2, 3]. Both ultrasound and MRI have been shown to be able to visualize Pacinian corpuscles in the hand [4, 5]. The higher inherent signal at ultra-high-field 7-T MRI can be exploited—among others—for higher spatial resolution [6]. This can be used to better depict the whole network of Pacinian corpuscles, focusing on the distribution, arrangement, and their exact location in relation to the cutis and deep soft tissues within the hands and feet, thereby correlating the various locations with their function as sensory receptors for vibration and proprioception [7].

In this case report, we present novel high-resolution 3D images at 7-T MRI, illustrating the detailed anatomy of the Pacinian corpuscles in the hand and feet, highlighting their excellent demarcation and exact location within the skin layers and soft tissues as well as the distribution patterns.

## Case reports

Written informed consent was given from both participants prior to the MRI examination.

### Case 1

During sequence optimization for musculoskeletal imaging on an ultra-high-field 7-T MRI system (MAGNETOM Terra, Siemens Healthcare, Erlangen, Germany), a 3D Dual Echo Steady State (DESS) sequence was tested for the foot and the hand in a 56-year-old healthy man using a 28-channel transmit/receive knee coil (Quality Electrodynamics LLC, Mayfield Village, OH), with the following acquisition parameters for both the foot and the hand: TR/TE: 7.5/2.5 ms, flip angle: 25°, field-of-view: 160 × 160 mm^2^, voxel dimensions: 0.36 mm isotropic, receive bandwidth: 507 Hz/Px, water-selective excitation, and coronal acquisition. DESS imaging was chosen because of (a) its T2 contribution to the image contrast weighting; (b) its lower specific absorption rate, compared to a 3D spin-echo sequence; (c) its reduced sensitivity to variations of magnetic susceptibility, than a fully balanced gradient-echo sequence; (d) its good encoding efficiency; and (e) its motion sensitivity, which provided comparatively good suppression of flowing blood.

**Fig. 1:**
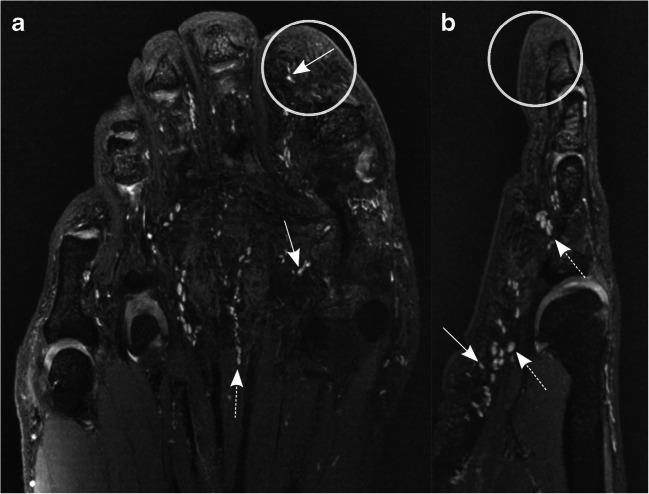
Coronal (**a**) and sagittal (**b**) 3D DESS images with partial maximum intensity projection (MIP) of the left forefoot of a 56-year-old asymptomatic male volunteer. Both images demonstrate numerous homogenously hyperintense ovoid nodules in the plantar subcutaneous fat (arrows) and along the deep muscle fascia (dashed arrows), mostly arranged in typical “chain-like” formations, representing Pacinian corpuscles. Interestingly, only few Pacinian corpuscles can be seen at the tip of the toes (circle)

**Fig. 2:**
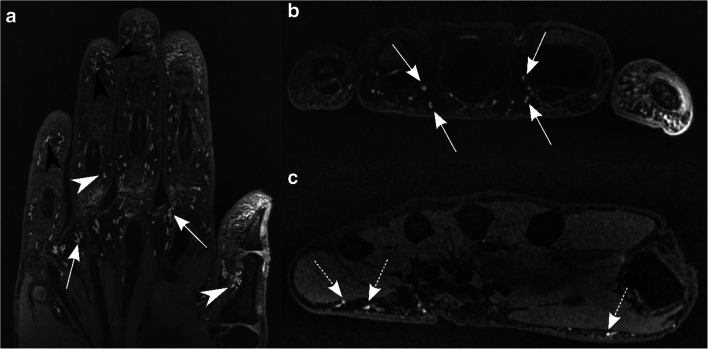
Coronal (**a**) and axial (**b**, **c**) DESS images of the left hand of a 56-year-old asymptomatic male volunteer. The axial images are from the level of the metacarpophalangeal joints (**b**) and the thenar/hypothenar (**c**). The network of Pacinian corpuscles—comprised of multiple hyperintense ovoid nodules—is illustrated in the palmar soft tissues, most tightly grouped at the level of the metacarpophalangeal joints (arrows), fingertips (black arrowheads), and proximal phalanges (white arrowheads). Only few Pacinian corpuscles are seen in the subcutaneous tissue of the thenar and hypothenar (dashed arrows)

This setup allowed identification of numerous hyperintense, sharply demarcated ovoid nodules within the plantar-sided soft tissues of the foot and toes and the palmar-sided soft tissues of the hand and fingers (Figs. 1 and 2). Regarding distribution, the mechanoreceptors seem to form a network, being arranged in chains and oriented along the palmar/plantar long axis of the digits with focal clusters around the metacarpophalangeal (MCP)/metatarsophalangeal (MTP) joints, the proximal phalanges, and the fingertips. Pacinian corpuscles were counted in the different locations by one fellowship-trained musculoskeletal radiologist: quantitative analysis revealed the highest numbers of Pacinian corpuscles at the plantar side of the MTP joints and proximal phalanges of the foot and the palmar side of the MCP joints, proximal phalanges of the hand, and fingertips (Table 1 and Fig. 5). Compared to the fingertips, fewer Pacinian corpuscles were seen in the plantar aspect of the distal phalanges of the toes. In the palmar aspect of the wrist, only few nodules can be seen. Diameters of the Pacinian corpuscles ranged from 1 to 5 mm.

**Table 1:** Pacinian corpuscle count. The table shows mean number of Pacinian corpuscles in the different localizations with range in parentheses for both healthy volunteers (*n* = 2)

		Digit I	Digit II	Digit III	Digit IV	Digit V
Hand	Distal phalanx	35.5 (30–41)	32.5 (24–41)	31.5 (28–35)	22 (22)	20.5 (20–21)
	Middle phalanx	*N.A.*	12.5 (10–15)	10.5 (8–13)	11 (10–12)	8.5 (8–9)
	Proximal phalanx	21.5 (18–25)	31.5 (28–35)	34 (34)	29 (28–30)	22.5 (20–25)
	MCP joint	22 (20–22)	26.5 (25–28)	26.5 (26–27)	25 (23–27)	25 (25)
	Metacarpal	4.5 (4–5)	4.5 (4–5)	5.5 (5–6)	3.5 (3–4)	5.5 (4–7)
Foot	Distal phalanx	12.5 (10–15)	11 (10–12)	11.5 (11–12)	12.5 (12–13)	11.5 (11–12)
	Middle phalanx	*N.A.*	5 (5)	6 (6)	5.5 (5–6)	4.5 (4–5)
	Proximal phalanx	23 (22–24)	21 (21)	22.5 (22–23)	21.5 (21–22)	21.5 (20–23)
	MTP joint	23 (22–24)	20.5 (19–22)	21 (19–23)	22.5 (22–23)	20.5 (20–21)
	Metatarsal	7.5 (7–8)	7.5 (6–9)	5 (5)	4 (4)	3 (2–4)

### Case 2

**Fig. 3:**
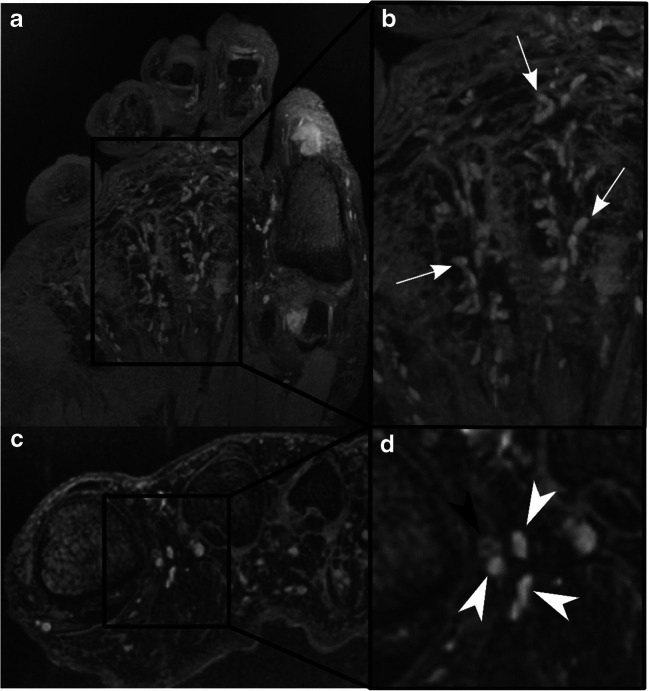
Coronal (**a**, **b**) and axial (**c**, **d**) DESS images of the left forefoot of a 34-year-old asymptomatic male volunteer. Axial images at the level of the proximal phalanges are shown (**c**, **d**). (**b**) and (**d**) illustrate magnified areas of (**a**) and (**c**), respectively. Pacinian corpuscles (arrows) are shown in the plantar subcutaneous fat and along the deep muscle fascia, mostly arranged in clusters. Cross section of the linear tubular-shaped blood vessels appears as central hypointense, and peripheral mildly hyperintense structures (black arrowhead) as opposed to homogenously hyperintense Pacinian corpuscles (white arrowheads); additionally, vessels were confirmed by following their linear, branching morphology over consecutive slices

**Fig. 4:**
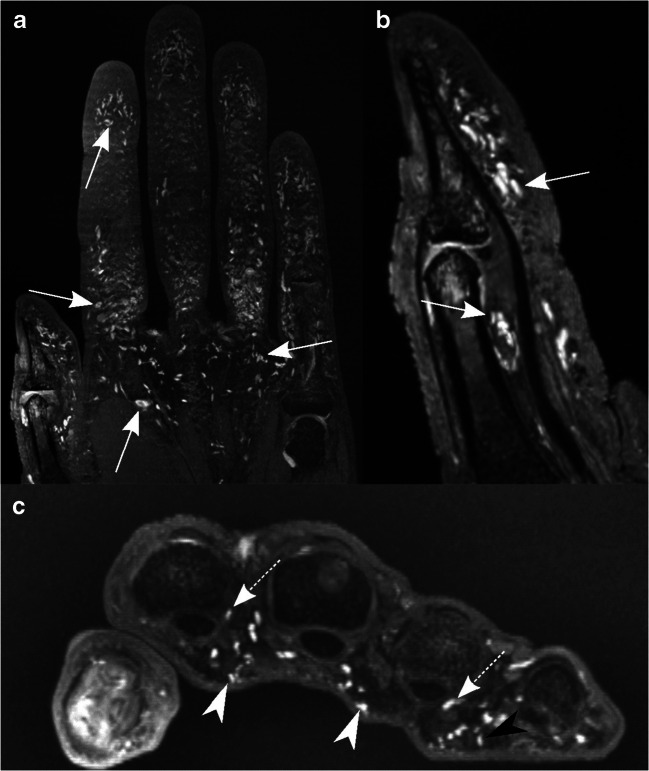
Coronal (**a**), sagittal (**b**), and axial (**c**) DESS images of the right hand of a 34-year-old asymptomatic male volunteer illustrate homogenously hyperintense ovoid-shaped nodules in the palmar-sided soft tissues. The sagittal image (**b**) shows the thumb. The axial image at the level of the metacarpophalangeal joints depicts Pacinian corpuscles in the subcutaneous tissue in contact with the dermal layer (white arrowheads), completely surrounded by subcutaneous fat (black arrowhead), or associated with connective tissues, e.g., tendons or the joint capsule (dashed arrows)

**Fig. 5:**
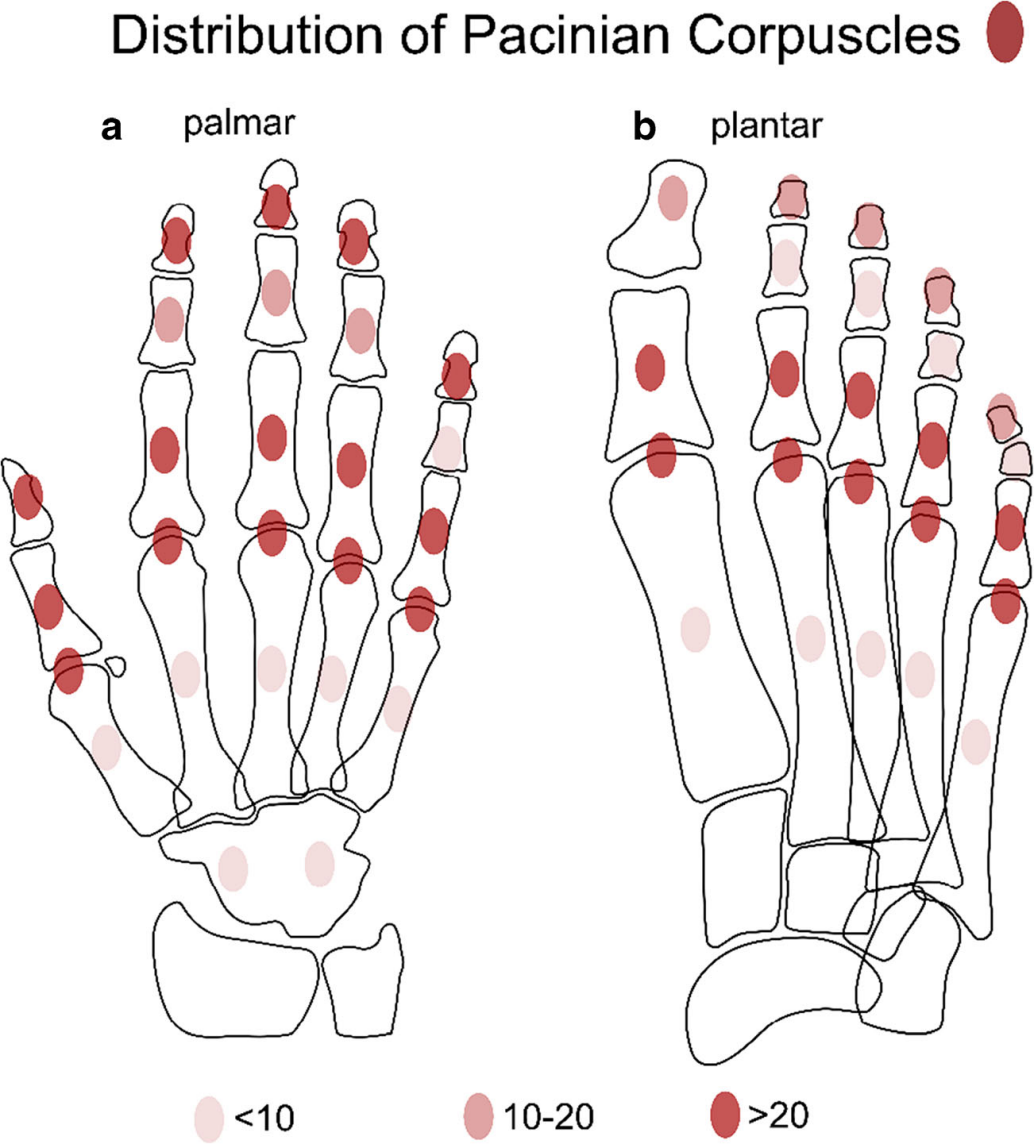
Schematic illustration of distribution pattern of Pacinian corpuscles in the palmar side of the hand (**a**) and plantar side of the foot (**b**). The quantity of corpuscles is indicated by the color intensity as shown in the figure legend: dark red represents areas with > 20 Pacinian corpuscles, light red marks areas with < 10 corpuscles. Locations with most tightly grouped Pacinian corpuscles are fingertips, proximal phalanges, and metacarpophalangeal/metatarsophalangeal joints. Only few Pacinian corpuscles were seen at the level of the phalanx media and the metacarpal/metatarsal bones and at the level of the wrist joint. Compared to the fingertips, fewer corpuscles were identified at the tip of the toes

**Fig. 6:**
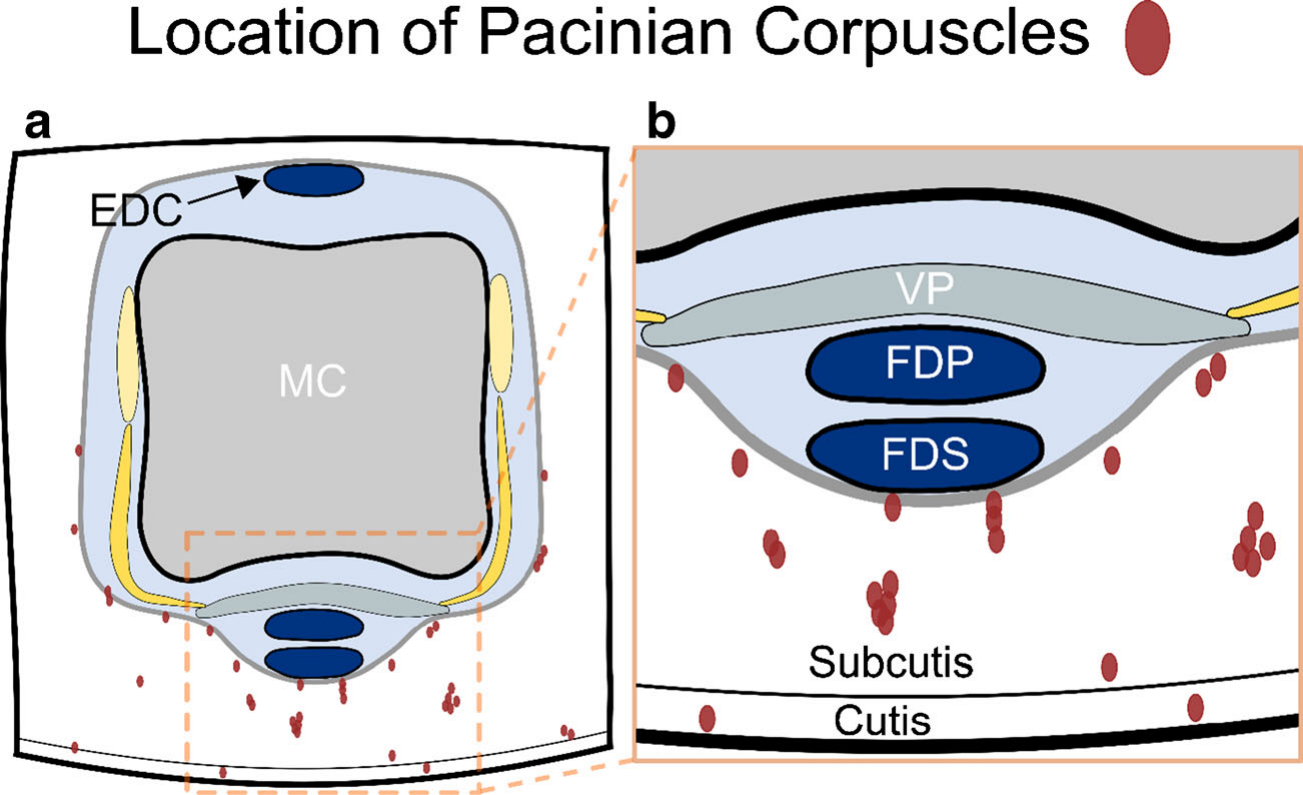
Schematic drawing illustrating the location of Pacinian corpuscles in relation to soft tissues: cross section through the proximal metacarpophalangeal joint (**a**) and magnified portion through the palmar side (**b**). Red ovoid ellipses represent Pacinian corpuscles, which can be located in the cutis and subcutis as well as in the deep soft tissues adjacent to tendons and the joint capsule. *EDC*, extensor digitorum communis; *FDP*, flexor digitorum profundus; *FDS*, flexor digitorum superficialis; *MC*, metacarpal; *VP*, volar plate

The same 3D DESS sequences as in case 1 were tested in a 34-year-old healthy man. Figures 3 and 4 illustrate the network of sharply demarcated hyperintense nodules in the sole of the foot, toes, the palm of the hand, and the palmar-sided soft tissues of the fingers. Identical to case 1, the nodules were most tightly grouped at the fingertips, the palmar aspect of the MCP joints, and proximal phalanges as well as clustered around the plantar side of the MTP joints and proximal phalanges (Fig. 5). As in case 1, two seemingly preferred distinct anatomical regions for the location of Pacinian corpuscles were identified: (1) cutaneous/subcutaneous and (2) deep in the soft tissues, adjacent to connective tissue, e.g., tendons or joint capsules (Figs. 4c and 6). Diameters of the corpuscles ranged from 1 to 5 mm. Two criteria were used to differentiate the Pacinian corpuscles from vessels: (1) the linear and branching morphology of vessels was ensured by following their course over consecutive slices and (b) central hypointensity with peripheral hyperintense rim of vessels on cross section as opposed to homogenously hyperintense Pacinian corpuscles (Fig. 3c, d).

## Discussion

This case report illustrates the detailed MR imaging anatomy of the complex network of Pacinian corpuscle mechanoreceptors in the feet and hands that can be visualized with 3D DESS imaging at 7 T.

High-resolution ultrasound has been shown to be useful in the identification of Pacinian corpuscles [4]. However, MRI has the benefit of giving an excellent overview of the whole area, and thereby may better illustrate the arrangement and distribution of these mechanoreceptors. The depiction of Pacinian corpuscles in the palms has already been demonstrated by Rhodes et al. using standard two-dimensional T1-weighted and T2-weighted sequences at 3-T MRI with histologic correlation [5]. They described the nodules as T2-hyperintense, T1-isointense (compared with skeletal muscle) punctate structures. Ultra-high-resolution 7-T MR images of a single finger allowed a sharp demarcation of single Pacinian corpuscles among other anatomical structures [7]. In our case report, we could further elaborate the MRI appearance of the Pacinian corpuscles and their distribution in three-dimensional space in the entire hand and foot. Our image analysis confirmed the results published by Rhodes et al. [5] with numerous and tightly grouped Pacinian corpuscles at the level of MCP joints. In addition, we found Pacinian corpuscles in similar numbers and arrangement also on the palmar side of the proximal phalanges, which is consistent with anatomic/histologic studies [8, 9] and may be explained by using ultra-high-resolution three-dimensional sequences (with a voxel size of 0.36 mm isotropic) in our study as opposed to two-dimensional sequences with 2–3-mm slice thickness by Rhodes, offering a better depiction of all corpuscles, which can be as small as 1 mm in diameter [10]. Our findings regarding the Pacinian corpuscle distribution in the fingertips confirm results by Laistler and colleagues depicting tightly grouped Pacinian corpuscles in this location [7]. Our quantification analysis revealed similar numbers of Pacinian corpuscles in the fingertips, palmar side of MCP joints, and proximal phalanges, whereas the lowest number of Pacinian corpuscles was seen at the level of the middle phalanges, which is consistent with anatomic reports [8]. Interestingly—compared to fingertips—fewer Pacinian corpuscles were noted in the plantar soft tissues of the toes, which has similarly been described in anatomic studies [9, 11]. This difference may reflect a better adaptation for touch and vibration in the fingertips compared to the toes, where detection of body-weight balance during the gait as primary demand of mechanoreceptors may require a smaller number of Pacinian corpuscles [9]. In the sole of the foot, we found Pacinian corpuscles most frequently and tightly arranged at the level of the MTP joints and proximal phalanges, identical to the findings in the hand.

Our images allow distinction of two anatomical locations of the Vater-Pacini corpuscles within the soft tissues, which is in concordance with findings in anatomic studies [8, 9, 11]: (1) in the cutaneous/subcutaneous layer—adjacent to the dermis or without relation to the dermis—presumably responsible for vibration/pressure and (2) deep in the soft tissues in contact with various parts of the connective tissues, e.g., tendons or the joint capsule—supposedly being crucial for proprioception. Additionally, our high-resolution 3D images are able to illustrate the whole network of the Pacinian corpuscles, forming “chain-like” clusters in areas where they are most tightly grouped (e.g., fingertips, proximal phalanges, and MCP/MTP joints) [5, 12].

Although various histopathology-based case reports have described pathologies associated with the Pacinian corpuscles—e.g., neuroma or painful hyperplasia and hypertrophy [13–15]—our case reports of two healthy volunteers aim primarily to illustrate the normal anatomy, location, and distribution of the mechanoreceptors, rather than suggest MRI as a primary diagnostic utility in these rare cases. Nevertheless—if needed—ultra-high-resolution MRI has the ability to provide the surgeon with an exact image of the detailed anatomy for preoperative planning.

Our report has limitations. First, the differentiation between Pacinian corpuscles and vessels is based solely on the appearance in one sequence, which could be alleviated by applying an additional MR angiography. Second, we did not provide histologic proof that the nodules depicted by MRI are in fact Pacinian corpuscles. However, neither application of intravenous contrast agent nor histologic correlation was an option in this case report of two healthy volunteers. Nonetheless, based on findings of previous reports with histologic correlation [5] and the clear depiction of their nodular appearance in our 3D MR images as opposed to linear branching vessels, we can confidently assume the correct identification.

In conclusion, with this case report, we further elucidated the normal distribution and arrangement of Pacinian corpuscles in various locations in the soft tissues of both the hands and feet using ultra-high-resolution 7-T MR images.

## Abbreviations

DESS: Dual Echo Steady State
MCP: Metacarpophalangeal
MTP: Metatarsophalangeal

## References

[1] Zimmerman A, Bai L, Ginty DD (2014). The gentle touch receptors of mammalian skin. Science..

[2] Kim AW, Rosen AM, Brander VA, Buchanan TS (1995). Selective muscle activation following electrical stimulation of the collateral ligaments of the human knee joint. Arch Phys Med Rehabil.

[3] Kholinne E, Lee H-J, Lee Y-M, Lee S-J, Deslivia MF, Kim G-Y (2018). Mechanoreceptor profile of the lateral collateral ligament complex in the human elbow. Asia Pac J Sports Med Arthrosc Rehabil Technol.

[4] Riegler G, Brugger PC, Gruber GM, Pivec C, Jengojan S, Bodner G (2018). High-resolution ultrasound visualization of Pacinian corpuscles. Ultrasound Med Biol.

[5] Rhodes NG, Murthy NS, Lachman N, Rubin DA (2019). Normal Pacinian corpuscles in the hand: radiology-pathology correlation in a cadaver study. Skelet Radiol.

[6] Bangerter NK, Taylor MD, Tarbox GJ, Palmer AJ, Park DJ (2016). Quantitative techniques for musculoskeletal MRI at 7 tesla. Quant Imaging Med Surg.

[7] Laistler E, Dymerska B, Sieg J, Goluch S, Frass-Kriegl R, Kuehne A (2018). In vivo MRI of the human finger at 7 T. Magn Reson Med.

[8] Stark B, Carlstedt T, Hallin RG, Risling M (1998). Distribution of human Pacinian corpuscles in the hand. J Hand Surg.

[9] Jin ZW, Cho KH, Xu DY, You YQ, Kim JH, Murakami G (2020). Pacinian corpuscles in the human fetal foot: a study using 3D reconstruction and immunohistochemistry. Ann Anat.

[10] Rhodes NG, Murthy NS, Lehman JS, Rubin DA (2018). Pacinian corpuscles: an explanation for subcutaneous palmar nodules routinely encountered on MR examinations. Skelet Radiol.

[11] Kim JH, Park C, Yang X, Murakami G, Abe H, Shibata S (2018). Pacinian corpuscles in the human fetal finger and thumb: a study using 3D reconstruction and immunohistochemistry. Anat Rec.

[12] Lang-Stevenson AI (1984). Induction of hyperplasia and hypertrophy of Pacinian corpuscles. BMJ..

[13] Roset-Llobet J, Domenech-Mateu JM (1991). Uncommon number and distribution of the Pacinian corpuscles in a human hand. J Hand Surg (Br).

[14] Rhode CM, Jennings WD (1975). Pacinian corpuscle neuroma of digital nerves. South Med J.

[15] Reznik M, Thiry A, Fridman V (1998). Painful hyperplasia and hypertrophy of pacinian corpuscles in the hand: report of two cases with immunohistochemical and ultrastructural studies, and a review of the literature. Am J Dermatopathol.

